# Pediatric B cell repertoires are enriched for naive clonal phenotypes and are shaped by distinct selection dynamics

**DOI:** 10.3389/fimmu.2025.1618234

**Published:** 2025-10-17

**Authors:** Thomas Hsiao, Areen Shtewe, Uri Hershberg

**Affiliations:** Department of Human Biology, Faculty of Natural Sciences University of Haifa, Haifa, Israel

**Keywords:** pediatric B cell repertoires, clonal selection, age-dependent repertoire development, negative selection, clonal architecture, germline retention, immune repertoire maturation, B cell receptor sequencing analysis

## Abstract

B cell development in early life sets the stage for how the adaptive immune system will function. Although little is known about B cell biology in children, especially from tissue-residing B cells, it has been previously reported that children generally possess more naive B cell repertoires with less somatic hypermutation and fewer expanded clones compared to adults. In this paper, we move beyond these findings by studying how clonal selection differs between children and adults from both blood and tissue-residing B cells. By integrating deep sequencing data of immunoglobulin heavy chains from genomic DNA from extracted tissues and blood of 15 children and 9 adults across multiple datasets, we demonstrate that B cell repertoires in children are not only less mutated, but also undergo reduced negative selection pressures. Mutated clones in children are less likely to possess a trunk compared to adults, but the relative proportion of mutated clones with trunks increases with age in children. This pattern is not observed in adults, where the fraction of mutated clones that possess a trunk has reached a steady state. Furthermore, clones in children are more likely to contain unmutated germline sequences, exhibit a greater number of viable internal nodes in their lineages, and experience less negative selection in the framework regions of their receptors. Overall, our findings show that B cell development is not merely the accumulation of mutations with age, but rather reflects a shift from flexible, broadly permissive repertoires in childhood to refined, stringently selected repertoires in adulthood. This difference in the structure of pediatric repertoires could underlie their enhanced ability to respond to novel antigens. Together, these findings provide new insights into how selection pressures shape immunity across the human lifespan.

## Introduction

The ontogeny of B cells in early life is thought to pave the way for how the adaptive immune system functions for the rest of one’s life, shaping lifelong susceptibility to autoimmune disorders and allergies, as well as the ability to eliminate infections later in life (1–8). The efficacy and specificity of the adaptive immune system also varies greatly between children and adults, which has been highlighted by the SARS-CoV-2 pandemic (9–13). Additionally, the biology of B cells differs significantly between blood and tissues (14). B cells from blood are mostly naive, whereas in tissues they become activated, undergo affinity maturation and clonal expansion (15–18). Therefore, our understanding of the biology of B cells and the selection pressures they experience is strengthened by using tissue-derived samples.

The observation that the efficacy of the immune system is age-dependent has been a major focus of B cell research. It has been shown that the total number of peripheral blood B cells is highest in children and young adults, before decreasing in late life (19, 20). Somatic hypermutation (SHM) levels have been reported to increase within the first six years of life and then remain stable throughout most of adulthood (3, 21, 22). Age-dependent B cell subtypes have also been a main area of interest. A naive-like subset of peripheral memory B cells (MBCs) has been identified that is prevalent in children, but absent in adults, which may partially explain why children are better at warding off novel infections (19).

However, studies involving pediatric subjects have been limited, and most have analyzed B cells only from blood due to the difficulty of obtaining tissue samples (3, 19–21, 23–25). Most research on pediatric B cells focuses on isotype frequencies and SHM levels. For example, it has been shown that in young children, naive IgM/IgD B cells predominantly develop into IgM MBCs through germinal center–independent pathways, while class-switched IgG MBCs arise via germinal center–dependent mechanisms (22). In that study, where the authors had to pool tissue samples from across individuals to have enough material for deep sequencing—and in most others involving children—tissue sampling was extremely limited, and the sequencing data were utilized mostly for analyzing B cell receptor (BCR) isotype frequencies.

**Figure 1:**
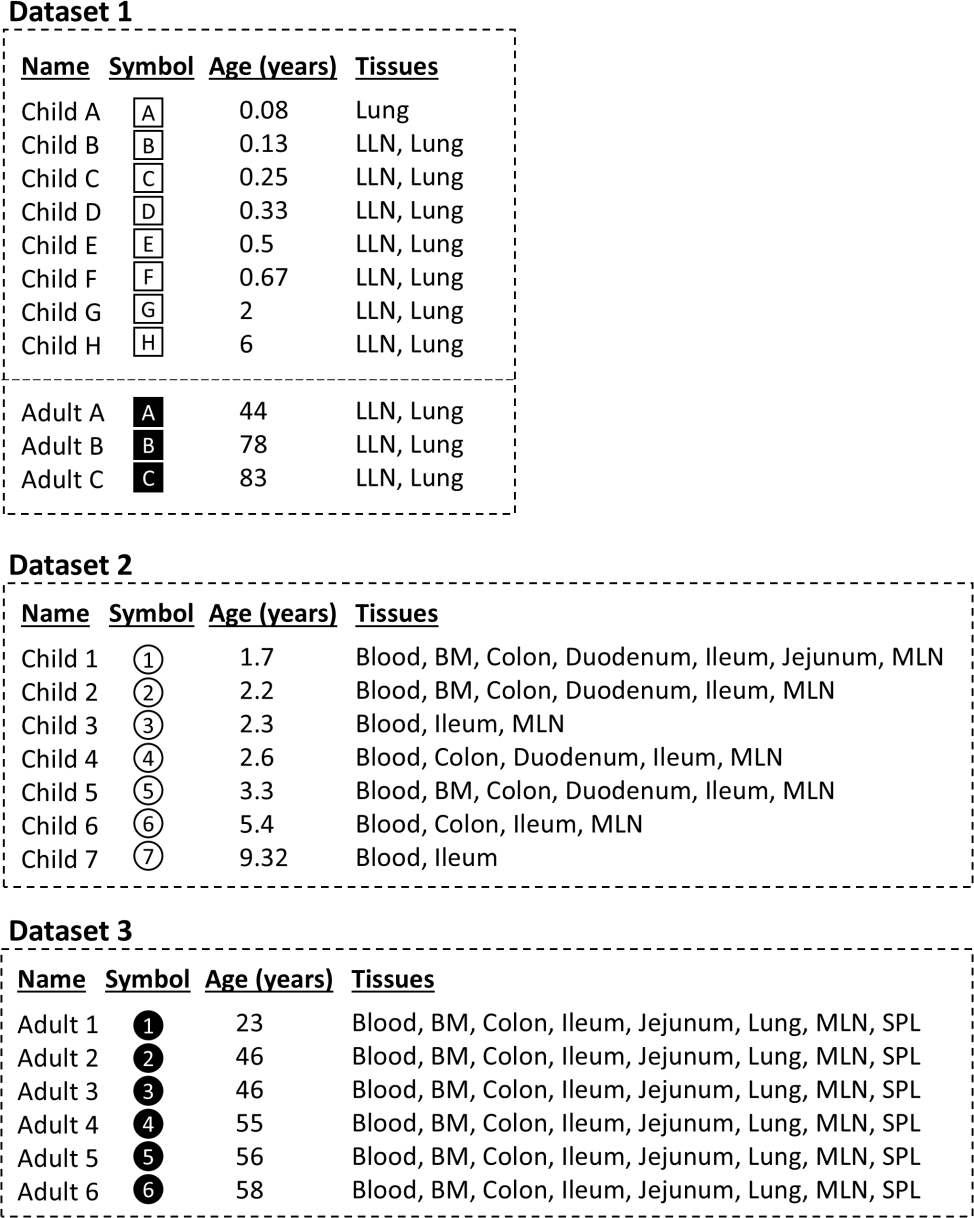
Individuals by dataset. Dataset 1 contains individuals with no known immunity-related issues. Dataset 2 contains children who have received intestinal transplants and were receiving immunosuppressants. Notably, all but one child are ≤ 2 years old in Dataset 1, while all but one child in Dataset 2 are > 2 years old. Dataset 3 contains adults with no immunity-related issues. Datasets 1 and 2 do not have any tissues that overlap, and therefore the individuals from these datasets are never aggregated in this study. Children have empty markers while adults have filled markers. BM, bone marrow; MLN, mesenteric lymph node; LLN, lung lymph node; SPL, spleen.

Consequently, more research is needed on pediatric B cell biology—especially on tissue-residing B cells—to better our understanding of these important early life events. Here, utilizing deep sequencing of immunoglobulin heavy-chain DNA from blood and multiple tissues across three datasets, we investigated the differences in B cell repertoire diversity between children and adults, with a particular focus on mutation and selection (**Figure 1**). To do so, we categorized B cell clones as either unmutated (fewer than five mutations present in ≥85% of sequences) or mutated (at least five mutations present in ≥85% of sequences). Mutated clones were further subdivided into trunk clones, defined as those sharing ≥5 identical mutations in ≥85% of sequences (indicative of a common selection event), and pre-trunk clones, which meet the mutation threshold but lack a shared set of mutations (**Supplementary Figure 1**). By comparing the relative abundance of these three categories, we were able to evaluate not only differences in overall mutation levels between children and adults but also differences in selective pressures shaping their repertoires.

Although it is known that both mutation frequency and clonality increase with age, it remains unclear whether B cell clones are subject to the same selective constraints throughout life. Using the clonal categories defined here, together with the analysis of non-synonymous to synonymous mutation ratios, we found that while the accumulation of mutations is continuous across age, the selection pressures experienced by B cells differ between children and adults. In early life, B cell clones display evidence of a more permissive and less constrained selection process.

## Results

### Children had highly naive B cell repertoires, whereas adult repertoires were mature

**Figure 2:**
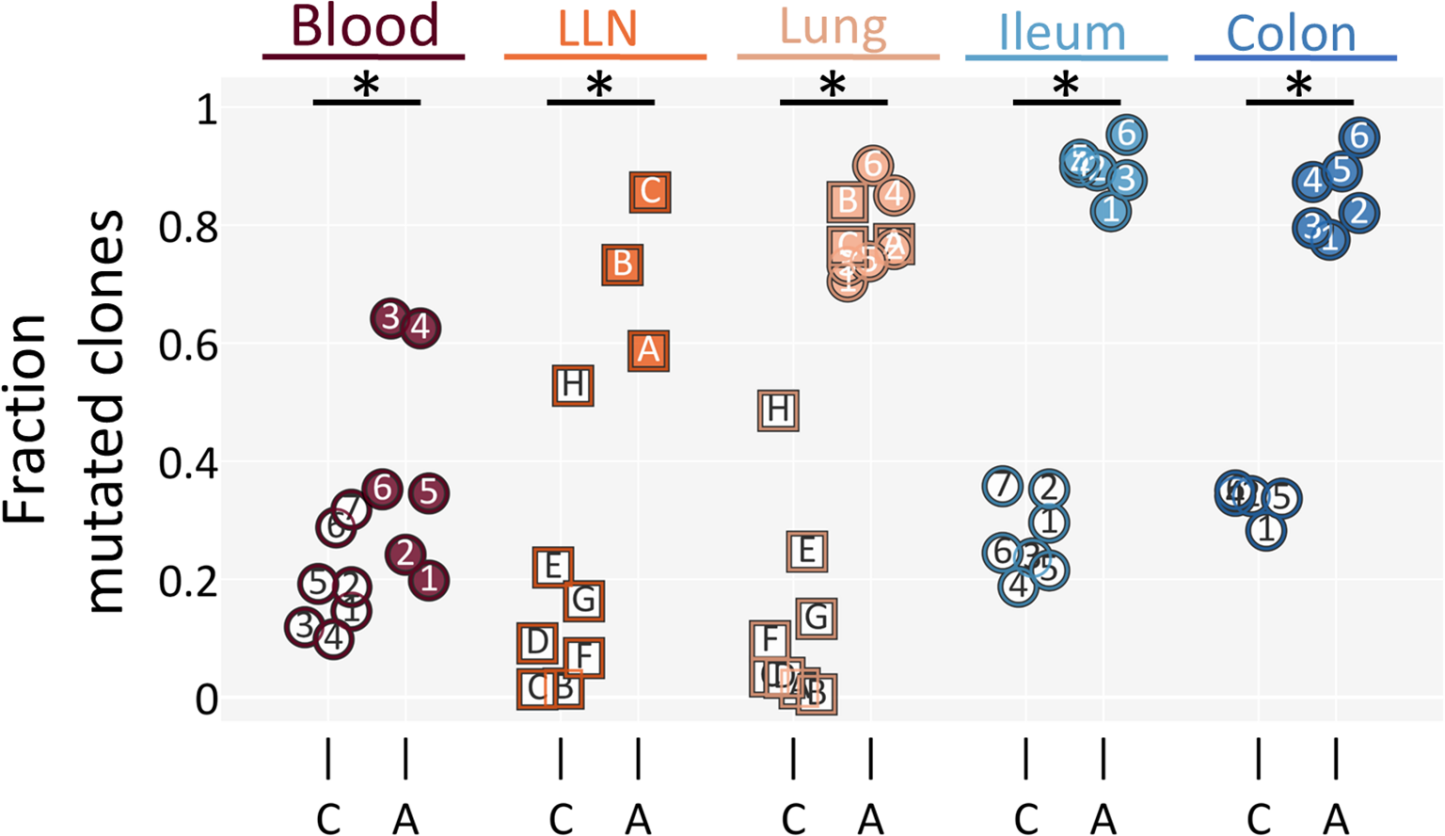
Tissue-residing clones exhibit significantly fewer mutations in children compared to adults. The fraction of clones that are mutated per tissue is shown. For each subject, the fraction of mutated clones out of total clones in a given tissue is shown. C - children; A - adults. The Mann-Whitney U test was used to test for significance, with p values < 0.05 denoted as * and p values ≥ 0.05 denoted as ns (not significant). Individuals are marked as in **Figure 1**. Clones were filtered for having 3+ nodes in their lineage. LLN, lung lymph node. Original names and sources for individuals can be found in **Supplementary Table 1**. Datapoints were filtered for having at least 10 clones.

**Figure 3:**
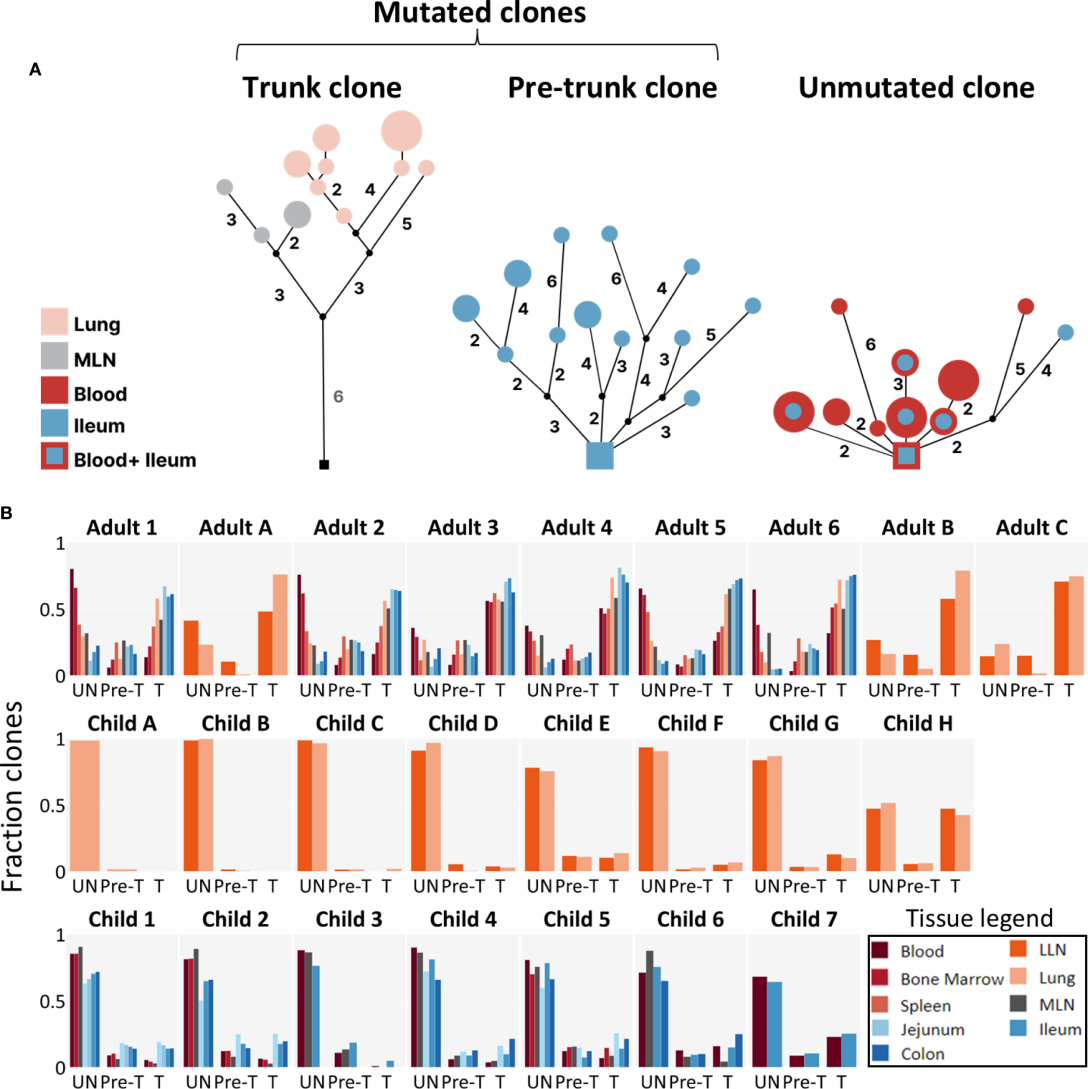
Repertoires from adults are dominated by trunk clones. **(A)** Representative lineages for a trunk clone, a pre-trunk clone and an unmutated clone. Multi-tiered clonal lineages exhibit different levels of common selection history for members in a clone. Most clones in adults have trunks, where all members share common mutations, while children have many pre-trunk mutated clones where members differ in their common mutation history. Lineages are rooted in the closest germline VH/JH gene allele in the IMGT database, marked with a square. Mutant types are marked with circles. Numbers indicate somatic mutations, unnumbered branches denote only one mutation between levels. Nodes are colored according to the tissue distribution of the sequence variants. Node sizes are proportional to sequence copy numbers. Black dots/squares indicate inferred nodes (see Methods). Each clone is identified by a unique VH/JH CDR3 combination. **(B)** The fraction of clones that are unmtuated (UN), pre-trunk (Pre-T) or trunk (T) is shown for each tissue. Clones were filtered for having 3+ nodes in their lineage. BM, bone marrow; MLN, mesenteric lymph node; LLN, lung lymph node; SPL, spleen.

We found that in all tissues (as well as in the blood) the fraction of mutated clones among all clones was dramatically lower in children than in adults (**Figure 2**). In fact, in contrast to what was observed in adult tissues, the fraction of mutated clones in pediatric tissues was so low that it resembled the levels observed in blood, which is known to be predominantly composed of naive B cells, even in adults (14). Furthermore, whereas nearly every mutated clone in adults contained a trunk—especially in tissue-residing clones—many mutated clones in children were pre-trunk clones and exhibited a more “bushy” shape (**Figure 3A**). Here, “bushy” refers to a lineage in which several branches emerge directly from the root node (as seen in the **Figure 3A** pre-trunk clone example), in contrast to the more “stalky” appearance of trunk clones, where typically a single branch extends from the root node. Lastly, among the small fraction of mutated clones that children have (aside from the two eldest children, who have a greater fraction of mutated clones among all clones), we observed an approximately even split between pre-trunk and trunk clones, a pattern observed across all tissues examined (**Figure 3B**). These findings suggest that most tissue-residing clones in children had not yet had sufficient time to accumulate mutations, either because they underwent fewer rounds of clonal activation or because they were subjected to frequent turnover.

### The fractions of mutated clones and trunk clones both increase with age, but not over the same developmental periods

**Figure 4:**
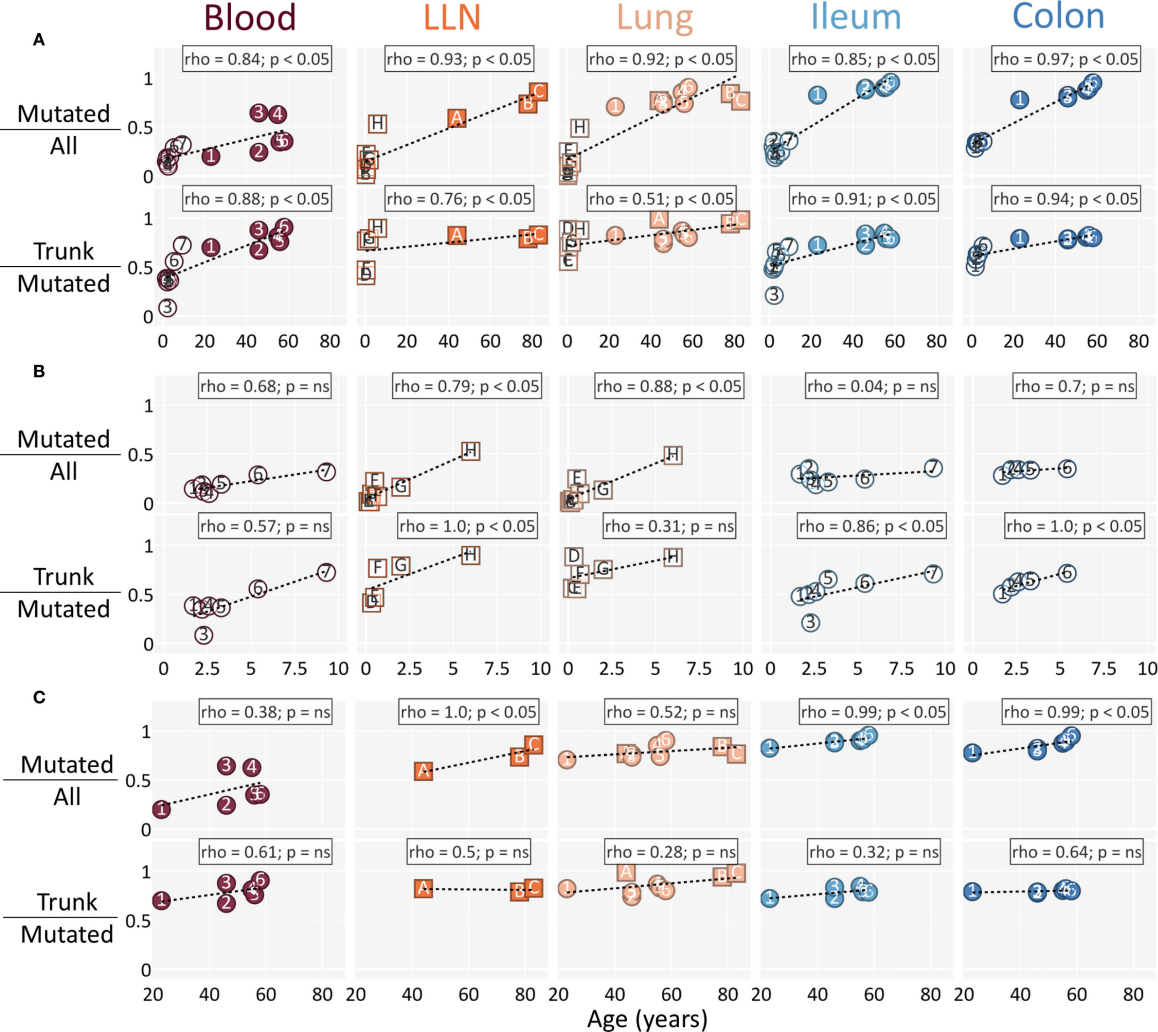
The fraction of trunk clones among mutated clones increases with age among children, but not adults. **(A)** Both children and adults are shown. **(B)** Only children are shown. **(C)** Only adults are shown. (Top row for **A**–**C**) The fraction of mutated clones among all clones per subject age is shown. (Bottom row for **A**–**C**) The fraction of trunk clones among mutated clones per subject age is shown. The dotted line represents the best fit line calculated by the least squares method. Statistics are calculated using Spearman’s rank correlation. Individuals are marked as in **Figure 1** and colored as in **Figure 2**. Clones were filtered for having 3+ nodes in their lineages. Datapoints were filtered for having at least 10 clones (datapoints that were omitted due to this threshold can be found in **Supplementary Table 3**). LLN, lung lymph node.

We found that in adults most mutated clones were trunk clones and few were pre-trunk clones, whereas in children the two categories were approximately evenly represented (**Figure 3B**). To address this difference, we examined how the fraction of mutated clones among all clones (mutated/all) and the fraction of trunk clones among mutated clones (trunk/mutated) changed with age. When children and adults were considered together, both measures showed a clear age-dependent increase (**Figure 4A**).

However, when children and adults were analyzed separately, the fraction of mutated/all clones increased with age in adults and in children with lung/LLN tissue samples (**Figure 1** Dataset 1), but no such trend was observed in children with blood and gut samples (**Figure 1** Dataset 2). In contrast, the fraction of trunk/mutated clones increased with age across all children but plateaued in adults, as evidenced by Spearman’s rank correlation, which showed a strong, significant positive correlation in children but only a weak, non-significant correlation in adults (**Figures 4B, C**, respectively). Notably, the results were unchanged when the oldest child in each category (Child H, Child 6, and Child 7, all over five years of age) was included among the adults, suggesting the fraction of trunk/mutated clones reached adult-like levels by five years of age (**Supplementary Figure 2**).

### Negative selection is less stringent in pediatric repertoires

**Figure 5:**
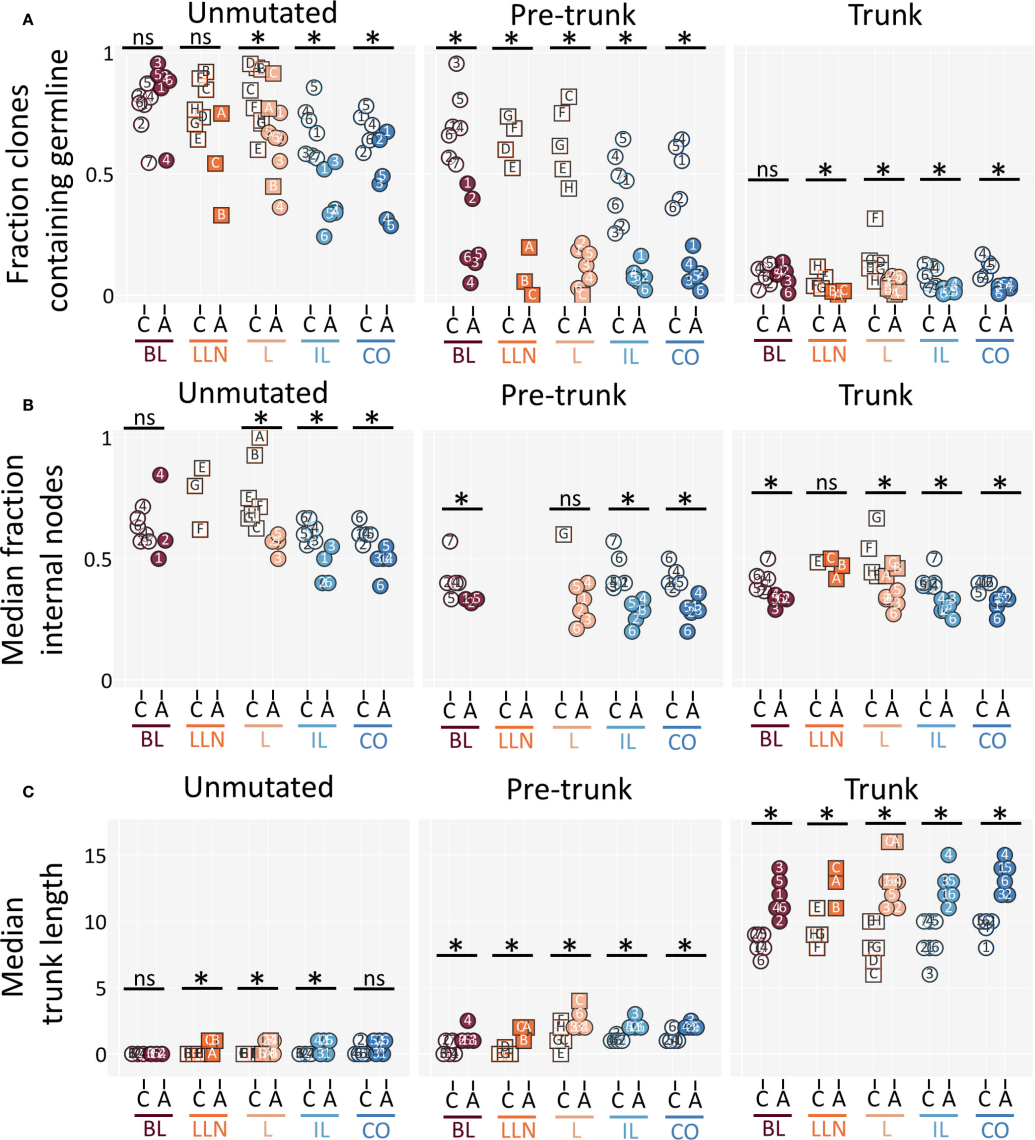
Repertoires in children are closer to a germline state. **(A)** Fraction of clones that contain a populated germline sequence (i.e., a surviving unmutated sequence – see Methods) for unmutated, pre-trunk and trunk clones. **(B)** The median value of the fraction of populated internal lineage nodes from total populated nodes per clone (as described in Methods). **(C)** The median trunk length for unmutated, pre-trunk and trunk clones is shown. (All panels) Individuals are marked as in **Figure 1** and colored as in **Figure 2**. Clones were filtered for having 3+ nodes in their lineages (except in **(B)** where clones were filtered with a stricter threshold of 5+ internal nodes; see Methods). Datapoints were filtered for having at least 10 clones (datapoints that were omitted due to this threshold can be found in **Supplementary Table 3**). C - child cohort; A - adult cohort; BL, blood; LLN, lung lymph node; L, lung; IL, ileum; CO, colo.

Clones in children appeared to expand under different selective constraints than those in adults. Across all clone categories and tissues, a greater fraction of clones in children retained observed germline sequences (even in highly mutated trunk clones), which is the consequence of completely unmutated clonal members surviving—an outcome rarely detected in adults (**Figure 5A**). In addition, pediatric clones were more likely to retain internal lineage nodes, which likely represent intermediary mutations that are suboptimal for antigen binding and are typically eliminated in adult clones (26, 27) (**Figure 5B**). Together, these findings indicate that clones in children experience weaker negative selection compared to adults.

Clones in children also exhibited shorter trunk lengths (or pre-trunk lengths for unmutated and pre-trunk clones) than their adult counterparts (**Figure 5C**). This difference likely reflects the fact that pediatric clones had less time and fewer antigenic exposures to accumulate sequential mutations. Overall, these results show that all clonal types in children lean toward a more naive state compared to those in adults, due to the combined effects of weaker negative selection and a shorter history of mutation accumulation.

### Framework regions experience less negative selection in children compared to adults

To assess the effects of positive and negative selection, we calculated the ratio of non-synonymous to synonymous mutations (replacement [R] to silent [S] mutations, respectively) among all clones for each tissue. In general, an R/S ratio near two indicates neutral selection; values above two reflect positive selection for amino acid substitutions that enhance antigen binding, while values below two reflect negative selection against deleterious substitutions (noting that codon usage biases in immunoglobulin genes can shift the expected neutral baseline from two) (28–30). Because complementarity-determining regions (CDRs) are generally subject to positive selection and framework regions (FWRs) to negative selection (31), we partitioned mutation counts within each clone accordingly.

**Figure 6:**
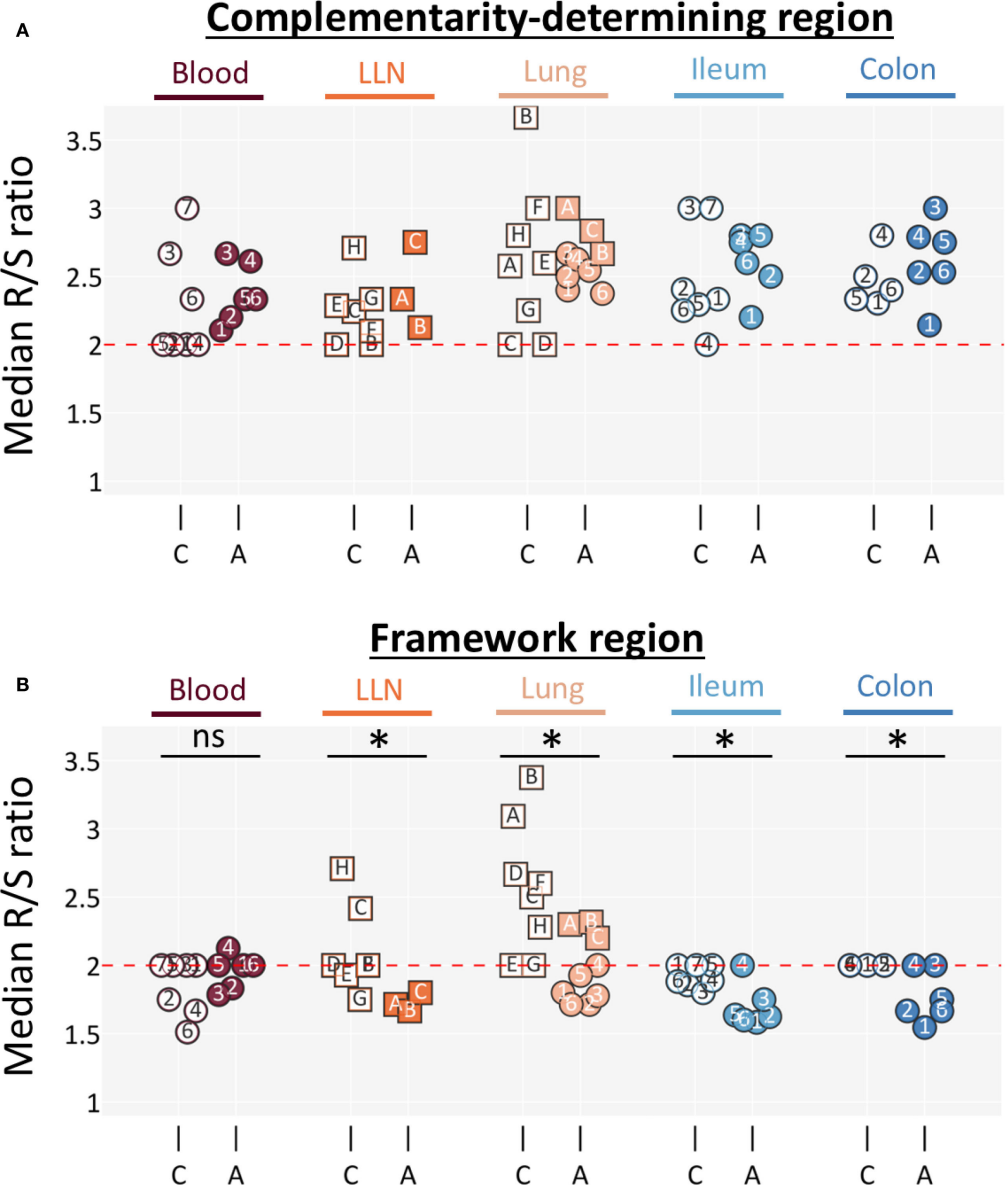
Framework regions exhibit less negative selection pressure in children. Median clonal R/S ratios is shown for **(A)** CDR and **(B)** FWR. The red dotted line highlights an R/S ratio of two, which is generally considered to represent neutral selection pressure. Clones were filtered for having 3+ nodes in their lineages. Datapoints were filtered for having at least 10 clones (datapoints that were omitted due to this threshold can be found in **Supplementary Table 3**). Individuals are marked as in **Figure 1** and colored as in **Figure 2**. LLN, lung lymph node; C - children; A - adults. An asterisk (*) indicates a significant difference (two-sided Mann-Whitney, p < 0.05). CDR groups showed no significant difference according to the Kruskal-Wallis test (p > 0.05) and were therefore not tested with the Mann-Whitney test.

Comparing the distributions of CDR R/S mutation ratios across clones, we found median values consistently above two in both children and adults, with no significant differences across tissues or age groups (Kruskal–Wallis, p ≥ 0.05) (**Figure 6A**). By contrast, the distributions of FWR R/S ratios differed significantly between children and adults (Kruskal–Wallis, p < 0.05). Across all tissues, median FWR R/S ratios were lower in adults than in children (two-sided Mann–Whitney, p < 0.05) (**Figure 6B**). Notably, in most adult tissues the median FWR R/S ratio dropped below two, consistent with stronger negative selection, whereas in pediatric tissues the ratios were close to two, reflecting reduced negative selection pressure on structural regions of BCRs.

## Discussion

Our study demonstrates that pediatric B cell repertoires are more germline-like and subject to distinct selection pressures compared to adults, revealing fundamental differences in how clonal lineages mature across the human lifespan. Children’s repertoires not only carry fewer mutations but also show weaker pruning of germline and intermediate sequences, reflecting a more flexible and less constrained state. By adulthood, repertoires become more structurally refined, with expanded trunks and reduced representation of intermediate members. Additionally, the methodology introduced here—grouping clones into unmutated, pre-trunk, and trunk categories—provides a novel framework for analyzing B cell repertoires, capturing distinctions in clonal maturation and selection that are not apparent from mutation frequency alone. Moreover, because this framework does not rely on isotype frequencies, it enables the study of repertoire dynamics even in datasets lacking isotype information, which is often the case in deep sequencing efforts that have sampling limitations.

Consistent with previous reports (3, 32–35), we found that B cell repertoires in children were dominated by unmutated clones, whereas adult repertoires were composed mostly of mutated clones. Extending these findings, we show that the proportion of trunk/mutated clones increases progressively throughout childhood but plateaus in adulthood. This pattern indicates that the establishment of trunk clones is primarily a developmental process of early life. These findings highlight distinct temporal dynamics in the development of B cell repertoires during childhood.

The absence of an age-dependent increase in mutated/all clones in children from Dataset 2 contrasts with what is observed in children from Dataset 1, where mutated/all clones do increase with age (**Figure 4B**). This discrepancy may have two explanations: first, that mutated clones accumulate rapidly during the first two years of life and then slows thereafter (of note, all but one child in Dataset 2 were older than two, while all but one child in Dataset 1 were younger than two), or second, that the immunosuppressive treatments received by the Dataset 2 children impaired clonal expansion and maturation. Given the consistency of patterns across multiple tissues and datasets, including those from non-immunosuppressed children, the more likely explanation is that mutated clones accumulate more rapidly in the earliest years of life rather than being substantially dampened by immunosuppression. This interpretation is also consistent with established evidence that infancy represents a period of rapid B cell expansion driven by extensive antigen exposure, after which the pace of accumulation slows (3). However, future work that focuses on this pediatric age range, particularly in individuals without immunity-related conditions or treatments, will be critical to disentangle these possibilities.

The continuous increase in trunk/mutated clones with age across all children indicates that the establishment of trunks continues throughout childhood and reaches adult-like levels by five years of age (**Figure 4C** and **Supplementary Figure 2**). The plateau observed in adults suggests that this process reaches a steady state once the memory repertoire has matured. Interestingly, these findings align with prior studies showing that early childhood marks a transition from germinal center–independent to germinal center–dependent maturation of MBCs, with memory repertoires resembling those of adults by approximately five to six years of age (22). Studies with broader sampling across early childhood—specifically, tissue sampling spanning more individuals and ages—will be essential to confirm this early window of rapid clonal change.

Beyond clone group dynamics, clonal structure also differed between children and adults. Adult clones typically possessed long trunks of shared mutations with fewer internal nodes. Pediatric clones, in contrast, frequently displayed a “bushy” structure in which mutated members branched close to the germline—often with unmutated germline-encoded B cells observed—and retained a greater number of intermediary internal nodes, even in trunk clones (**Figures 5A-C**). These internal sequences are thought to represent suboptimal mutations that do not sufficiently improve antigen binding and are usually eliminated through negative selection (26, 27). Their persistence in children, together with the survival of unmutated germline members, indicates that negative selection is less stringent during childhood. At the level of R/S mutation ratios, we found clear age-related differences in the strength of negative selection. Across all tissues, R/S ratios in CDRs were similarly elevated above two in both children and adults, consistent with sustained positive selection. In contrast, FWRs displayed significantly lower R/S ratios in adults, with median values often below two, consistent with stronger negative selection against destabilizing mutations in structural regions. In children, FWR R/S ratios remained closer to neutral levels (**Figure 6**). Together, these results indicate that pediatric repertoires undergo less rigorous pruning and maintain a more varied and less refined set of clonal variants.

Trunk length provided a complementary measure of repertoire maturation. Trunks (and pre-trunks in unmutated and pre-trunk clones) were consistently shorter in pediatric clones than in adult clones, a difference best explained not by weaker positive selection but by limited time and antigenic exposure to accumulate sequential mutations. Taken together, these observations demonstrate that pediatric repertoires are not simply less mutated versions of adult repertoires. Rather, they are shaped by distinct selective dynamics: weaker negative selection, shorter trunks reflecting limited exposure history, and permissive maintenance of germline and intermediary variants. This structural “naivety” may help explain why children often mount more effective responses to novel pathogens (particularly viral infections), as repertoires closer to a germline state may retain broader antigen-recognition potential (36). These differences also provide a potential mechanistic basis for why children often respond robustly to vaccines and new infections, whereas adults rely more heavily on established memory compartments.

In conclusion, we provide evidence that B cell repertoires and their underlying selection processes differ markedly between children and adults, offering insight into why children may be better equipped to respond to novel infections. Pediatric repertoires differ from adult repertoires not only in overall mutation burden but also in how selection operates within clones. In childhood, clones accumulate trunks while still retaining germline and intermediate members due to weaker negative selection. By adulthood, these processes reach a steady state, yielding repertoires that are more mutated, structurally pruned, and less flexible in antigen recognition. Overall, these findings highlight the importance of analyzing tissue-resident B cells and underscore the need for systematic sampling across childhood to identify the precise developmental windows when selective pressures shift. Future studies spanning more individuals, ages, and tissues will be essential to corroborate these findings and expand our understanding of how age-dependent dynamics of repertoire selection shape immunity across the human lifespan.

This study is not without limitations. As a meta-analysis spanning three major efforts to deeply sequence tissue-resident B cell repertoires (14, 34, 35), it is constrained by the design of those original datasets. We cannot retrospectively query B cell isotypes or subtypes that are not specified in the source studies, nor can we optimize age stratification beyond what was originally reported. Nevertheless, the strength of these datasets lies in their sequencing depth, which enabled us for the first time to compare selection dynamics across both tissues and individuals without conflating these distinct systems.

To mitigate the lack of isotype- and subtype-level information, we grouped clones into broad functional categories: unmutated (naive), pre-trunk (mutated but without a shared trunk), and trunk clones (mutated with shared trunks). While this classification is coarse, it captures the key differences in mutation history and selective pressures that are central to our analysis.

Two other potential limitations are age distribution across the individuals and the immunosuppressive treatments taken by the children from Dataset 2. Regarding pediatric age range, the age groups ≤2 years and >2 years were mostly analyzed separately. In the Dataset 2 cohort (n = 7), only one child was under two years of age (Child 1, 1.7 years old), while in the Dataset 1 cohort (n = 8), only one child was over two years of age (Child H, age 6). Thus, at most a single child ≤2 years of age was compared with children >2 years of age within a given analysis, or vice versa. Furthermore, it is worth noting that Adult 1 was younger than the other adults (23 years old versus the other adults who were 46–58 years of age). Regarding immunosuppression, all pediatric blood and gut samples (Dataset 2) were obtained from transplant recipients taking immunosuppressive treatments, raising the possibility that clonal expansion and maturation could have been impaired. By contrast, lung and lymph node (LLN) samples (Dataset 1) were from non-immunosuppressed individuals. Importantly, these two groups did not overlap in tissue type: immunosuppressed children contributed blood and gut samples, while non-immunosuppressed children contributed lung and LLN samples, meaning the children from these two datasets were never aggregated within an analysis, since each tissue was always plotted separately. Full details are provided in **Figure 1** and **Supplementary Tables 1** and **2**, which also indicate each individual’s age, sex, tissue type, and immunosuppression status.

These factors—immunosuppressive treatment history and age differences—could certainly influence individual repertoires, and we acknowledge them as important considerations. To mitigate their impact, we deliberately avoided pooling samples or relying on summary statistics, and instead presented each individual as its own datapoint in all figures. This strategy preserves individual-level variability and makes it possible for readers to see how immunosuppression, age, or immune history may contribute to differences across subjects.

While these sources of variability cannot be fully disentangled in our study, children from the two groups were never aggregated in the same analysis, and the overall patterns—including elevated R/S ratios in CDRs and V and J gene usages—were consistent across all individuals, including the immunosuppressed children. Furthermore, we checked and compared V and J gene usage as an additional metric of overall repertoire structure (**Supplementary Figure 3**). As expected, certain V and J genes were preferentially used, reflecting known biases in recombination and repertoire assembly; however, these preferences were consistent across children and adults. We observed little evidence of systematic differences between groups, and importantly, the immunosuppressed children in Dataset 2 displayed usage patterns closely aligned with those of other individuals. This corroborates our claim that the immunosuppressive treatments in this cohort did not introduce detectable biases into the repertoire features analyzed here.

Despite its limitations, our study provides one of the first systematic comparisons of pediatric and adult B cell repertoires across multiple human tissues. Given the scarcity of pediatric B cell sequencing data, these results offer rare insights into the selective forces shaping immune development in early life. Future studies with larger cohorts spanning a broader range of ages, and ideally excluding individuals with immunity-related conditions, will be essential to refine and extend our findings. The caveats described above highlight the interpretive boundaries of our work. Nonetheless, the consistency of observations across tissues, age groups, and datasets supports the robustness of our conclusions that pediatric immune repertoires are shaped by distinct selective dynamics—characterized by weaker negative selection and greater permissiveness toward germline and intermediary variants.

## Methods

### B cell data

To compare the B cell landscape between children and adults, we analyzed bulk heavy chain B cell sequencing data from three previous studies of tissue and blood B cell repertoires in humans (**Figure 1** and **Supplementary Tables 1** and **2**). As previously reported, six adults were sequenced in peripheral blood lymphocytes (PBL), bone marrow, lung, spleen, mesenteric lymph nodes (MLN), ileum, jejunum and colon (14) (Dataset 3 from **Figure 1**). Eight children and three adults were sequenced in lung and LLN (35) (Dataset 1 from **Figure 1**). Lastly, seven children were sequenced in ileum, colon, jejunum and blood (34) (Dataset 2 from **Figure 1**). These seven children received gut tissue transplants and were taking immunosuppressants. In some of these children from Dataset 2, a small subset of their samples displayed signs of rejection (as indicated in the sequencing metadata, determined by accompanying histology analyses of the samples in the original study) (34). In an effort to reduce any complications associated with rejection, we excluded all clones associated with rejection: each clone was associated with a list of sample IDs in which it was detected; if any of those samples had a rejection level greater than zero (based on histology analysis of the sample), the clone was removed from all analyses—even if it also appeared in different samples with a rejection level of zero. Therefore, only clones exclusively from samples with a rejection level of zero, which represented the majority of cases, were retained for analysis.

### Sequence annotation

In the three datasets analyzed, immunoglobulin heavy chain rearrangements were amplified directly from genomic DNA extracted from tissues or blood and all sequencing data were stored and annotated with ImmuneDB (37) as in their original studies (14, 34, 35). Because B cell clones accumulate mutations, the unique sequences belonging to a single clone are not identical at the nucleotide level, but instead represent related variants derived from the same initial germline V(D)J rearrangement. Specifically, to annotate all sequences to the appropriate clones, each sequence was assigned to clones in which all sequences shared the same VH and JH genes, had identical CDR3 amino acid length, and had at least 85% CDR3 amino acid identity. Notably, our analyses used two clonal annotations provided by ImmuneDB: 1) the clone_stats table listing all unique mutations in a clone by their sequence prevalence and 2) the clones table which lists the lineages constructed for each clone by neighbor-joining. These lineages were divided into sequenced nodes representing all the unique sequences in a clone, and inferred nodes, which represent missing common ancestors and are inferred from patterns of sequenced mutations. Each clonal lineage was rooted in an inferred germline that in some clones was also observed in the sequenced B cell receptors.

### Differentiating between populated and non-populated nodes

Some nodes within a clonal lineage are inferred based on the presence of their descendants. If two or more sequences share some—but not all—mutations, we infer an intermediate common ancestor sequence/node that carries the shared mutations. Additionally, each clone has an inferred germline sequence associated with it, as described above in the sequence annotation section. We refer to a germline node as “populated/observed” if we identified an actual B cell receptor sequence in the data that is identical to the inferred germline sequence.

### Clone filtering and sharing across tissues

Many of our analyses measured the complexity of clonal lineages. Therefore, only clones with at least 3 sequenced nodes (inferred nodes do not count) in their lineages were considered in our study. For **Figure 5B**, we applied a stricter filter of ≥5 internal nodes. This ensured that clones had enough lineage complexity for meaningful analysis, while avoiding overly stringent thresholds that would exclude too many clones. Datapoints in the analyses were filtered for having at least 10 clones (most datapoints had hundreds or thousands of clones) as datapoints with fewer than 10 clones are unlikely to yield reliable results. Although the threshold of 10 is somewhat arbitrary, it provides a practical balance between maximizing data inclusion and avoiding over-interpretation of datapoints based on too few clones. Clones were considered to be in a tissue if they contained at least one unique sequence from that tissue. To elaborate, a clone is defined as a group of sequences (each derived from an individual B cell) that originate from the same initial V(D)J germline rearrangement (along with additional criteria as described in the **Methods** under **sequence annotation**) and differ slightly due to somatic mutations acquired over the clone’s lifetime. As a clone expands, its progeny may accumulate mutations and migrate to different tissues. Consequently, sequences from the same clone may be detected in multiple samples, including those from different tissues. Thus, if a sequence from a clone was detected in a blood sample, we classified that clone as present in blood. When sequences from a clone were detected in samples from more than one tissue, the clone was considered to be present in each of those tissues. Lastly, for the children who received intestinal transplants (Dataset 2), allograft and native tissues were combined (e.g., ileum and ileum allograft were both treated as ileum), and samples were not divided by time points. All individuals/datapoints were included in the graphs for their corresponding category, except when a datapoint was omitted due to having fewer than 10 clones (**Supplementary Table 3**). For instance, a given child with ileum can be assumed to be included in the “child ileum” category for a given graph, unless that child is listed in **Supplementary Table 3** for the given graph (which lists datapoints that were omitted due to having fewer than 10 clones).

### Division of clones into unmutated, pre-trunk and trunk clonal groups

To assess clonal structure complexity, we used the presence of a *trunk*—a set of shared mutations across sequences within a clone—as a defining feature. We defined a trunk in a clone as a shared set of at least five unique mutations present in ≥85% of the clone’s sequences, a threshold that is highly unlikely to occur by chance and thus reflects a common selection event for the lineage (38). Based on this criterion, we classified clones into unmutated and mutated categories. Furthermore, we subdivided the mutated clones into pre-trunk clones and trunk clones (**Supplementary Figure 1**):

- Unmutated clones: <5 mutations in ≥85% of sequences.
- Mutated clones: ≥5 mutations in ≥85% of sequences.
  - Pre-trunk clones: mutated clones that do not meet the trunk criterion (i.e., <5 shared mutations across the clone).
  - Trunk clones: mutated clones with ≥5 shared mutations present in ≥85% of sequences.

To ensure that the clone lineage structure reflected the type of selection the clone had undergone only clones containing at least three observed nodes (as opposed to inferred nodes; see **differentiating between populated and non-populated nodes in Methods**) in their lineage were analyzed.

### Ways of counting clone and sequence numbers

*Unique seq count:* All sequences within a clone with the exact same mutations are grouped as a single unique sequence. For example, if a clone has 100 cells carrying the same sequence, it is represented once as a unique sequence with a copy number of 100. *Copy number count*: The number of times a unique sequence was detected in a sample. While sequencing errors can affect this value, in general each B cell contributes one copy. Here, the copy number count is the sum of copy numbers across all samples for an individual. *Sample count:* The number of sequencing samples obtained for an individual. Each sample corresponds to a sample of genomic DNA from blood or tissue used for sequencing. *Clone count:* The total number of unique clones (as defined in **sequence annotation** in **Methods**) identified across all samples for an individual.

### Statistical analysis

Nonparametric tests were used throughout as described in the results and figure captions with a p-value cutoff of (p < 0.05) to indicate significance.

## Data Availability

The original contributions presented in the study are included in the article/**Supplementary Material**. Further inquiries can be directed to the corresponding author.

## Supplementary material

Supplementary Figure 1 Schema of clone group definitions and division by mutation level and number of shared mutations.

Supplementary Figure 2 (Top) The fraction of mutated clones among all clones per age for adults and the oldest child from each tissue is shown. (Bottom) Shown is the fraction of trunk clones among all mutated clones per subject age, for the same individuals as in the top panel. The dotted line represents the best fit line. Statistics are calculated using Spearman’s rank correlation. Individuals are marked as in **Figure 1** and colored as in **Figure 2**. Clones were filtered for having 3+ nodes in their lineages. Datapoints were filtered for having at least 10 clones. LLN, lung lymph node.

Supplementary Figure 3 V and J gene usage. The fraction of V and J gene usage among clones is shown. Datapoints were filtered for having at least 10 clones. For each individual, V and J genes accounting for less than 1% of their repertoire in a given tissue were excluded. Then, a gene was retained only if it was observed in at least 3 children and 3 adults, or if its frequency exceeded 5% in any individual. This filtering was performed in order to make space in the figure by removing genes with very low usage. Individuals are marked as in **Figure 1**. Children are colored as in **Figure 2** while adults are colored with black markers.

Supplementary Table 1 Subject names, ages, original sources and immunosuppression status.

Supplementary Table 2 Subject sequencing and tissue data. BM, bone marrow; MLN, mesenteric lymph node; LLN, lung lymph node; SPL, spleen. In the original datasets, blood is called PBMC or PBL.

Supplementary Table 3 Datapoints omitted from figures due to having fewer than 10 clones. **Figure 5B** has an additional layer of filtering due to filtering clonal lineages for having at least five populated internal nodes, which is a strict filter (necessary to prevent results from being skewed by simple lineages) and results in many datapoints being omitted due to having fewer than 10 clones. LLN - lung lymph node.
