# Supplementary material for: Pediatric B cell repertoires are enriched for naive clonal phenotypes and are shaped by distinct selection dynamics
Source: Front Immunol. 2025 Oct 17;16:1618234. doi: 10.3389/fimmu.2025.1618234 (PMC12575387; doi:10.3389/fimmu.2025.1618234)
Supplement: Supplementary Figure 1 — Schema of clone group definitions and division by mutation level and number of shared mutations. [file DataSheet1.pdf]

Pediatric B cell repertoires are enriched for naive clonal phenotypes and are shaped by distinct selection dynamics

Thomas Hsiao[1], Areen Shtewe[1], and Uri Hershberg[1*]

[1]Department of Human Biology, Faculty of Natural Sciences University of Haifa, Haifa, Israel

*Correspondence: uri@sci.haifa.ac.il

SUPPLEMENTAL MATERIALS

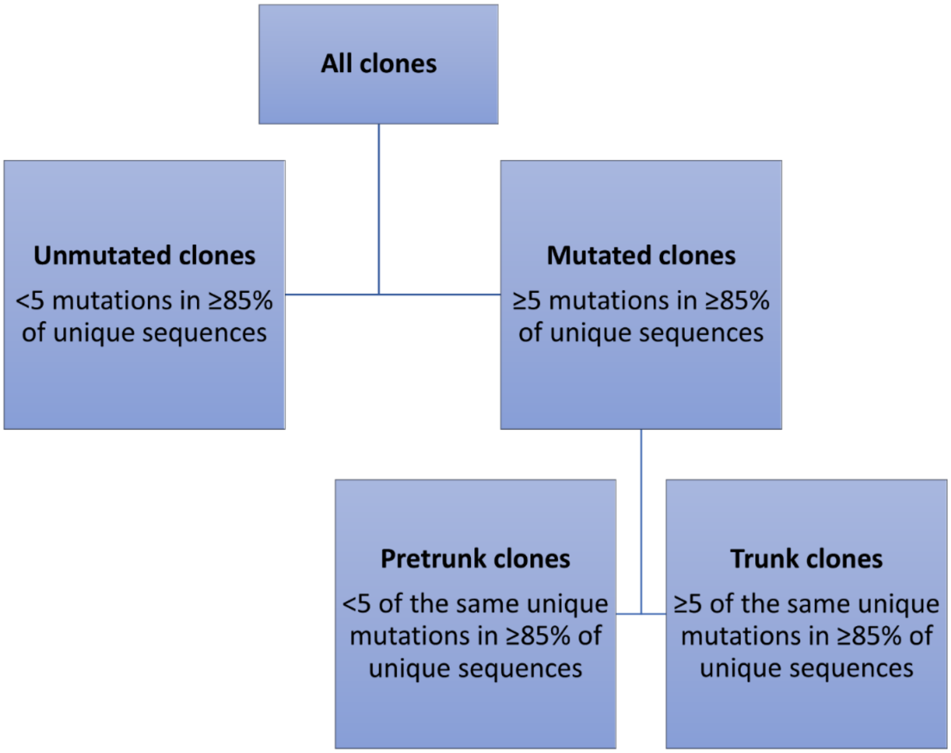

Supplemental Figure 1. Schema of clone group definitions and division by mutation level and number of shared mutations.

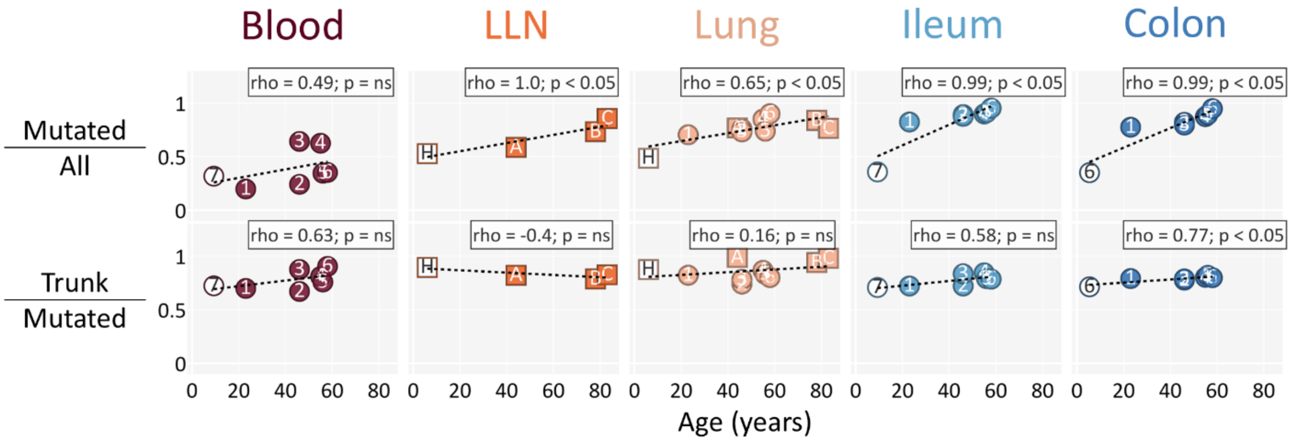

Supplemental Figure 2. (Top) The fraction of mutated clones among all clones per age for adults and the oldest child from each tissue is shown. (Bottom) Shown is the fraction of trunk clones among all mutated clones per subject age, for the same individuals as in the top panel. The dotted line represents the best fit line. Statistics are calculated using Spearman's rank correlation. Individuals are marked as in Figure 1 and colored as in Figure 2. Clones were filtered for having 3+ nodes in their lineages. Datapoints were filtered for having at least 10 clones. LLN, lung lymph node.

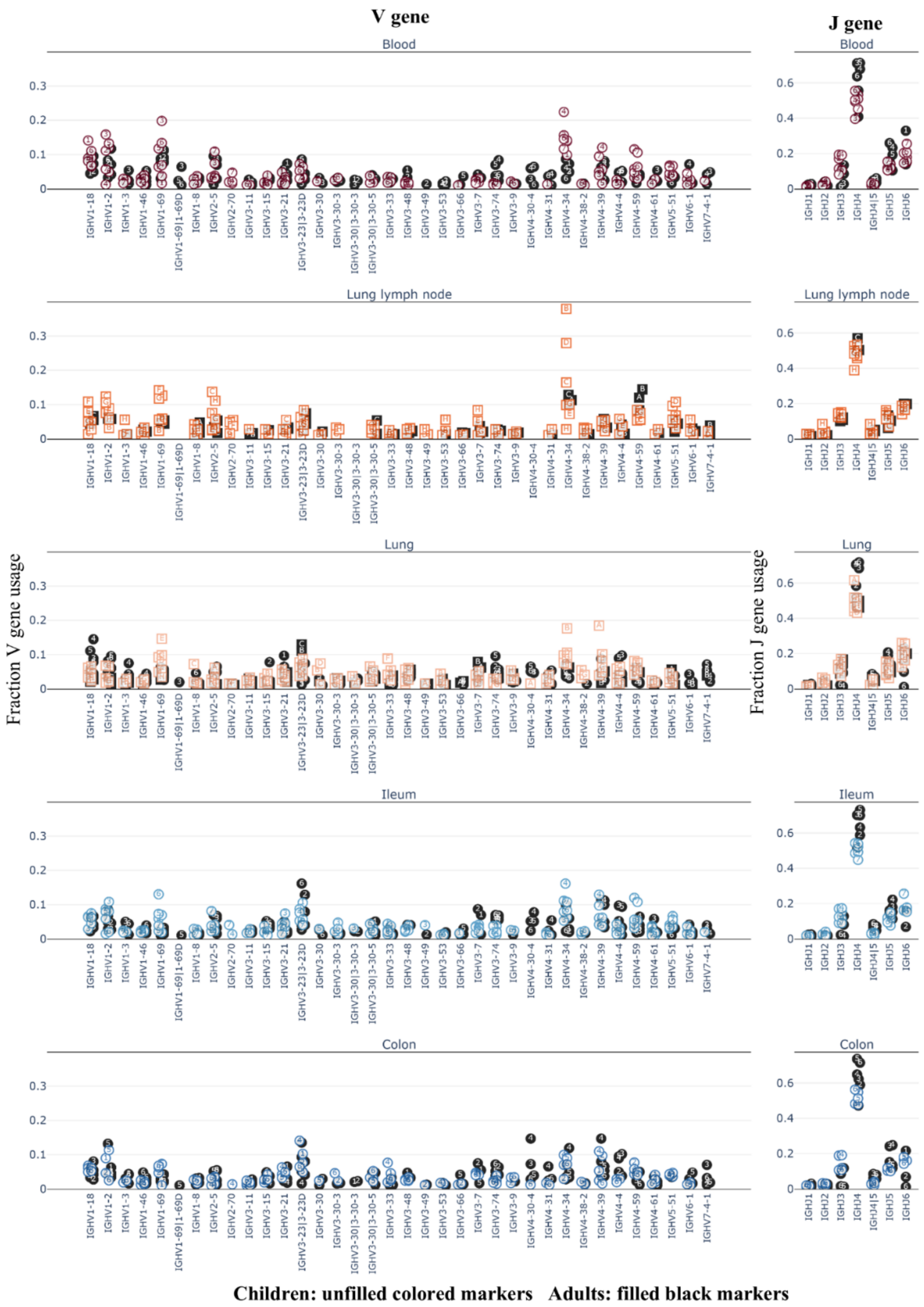

Supplemental Figure 3. V and J gene usage. The fraction of V and J gene usage among clones is shown. Datapoints were filtered for having at least 10 clones. For each individual, V and J genes accounting for less than 1% of their repertoire in a given tissue were excluded. Then, a gene was retained only if it was observed in at least 3 children and 3 adults, or if its frequency exceeded 5% in any individual. This filtering was performed in order to make space in the figure by removing genes with very low usage. Individuals are marked as in Figure 1. Children are colored as in Figure 2 while adults are colored with black markers.

Name	Name in original source	age (years)	Original source	Taking immune suppressants	Sex
Child A	HDL100	0.08	Matsumoto, R. *et al.*	No	Male
Child B	HDL155	0.13	Matsumoto, R. *et al.*	No	Male
Child C	HDL122	0.25	Matsumoto, R. *et al.*	No	Female
Child D	HDL134	0.33	Matsumoto, R. *et al.*	No	Female
Child E	HDL110	0.5	Matsumoto, R. *et al.*	No	Female
Child F	HDL104	0.67	Matsumoto, R. *et al.*	No	Female
Child G	HDL111	2	Matsumoto, R. *et al.*	No	Male
Child H	HDL116	6	Matsumoto, R. *et al.*	No	Female
Adult A	HDL471	44	Matsumoto, R. *et al.*	No	Male
Adult B	HDL410	78	Matsumoto, R. *et al.*	No	Female
Adult C	HDL423	83	Matsumoto, R. *et al.*	No	Male
Child 1	Pt20_R	1.7	Fu, J. *et al.*	Yes	Female
Child 2	Pt23_R	2.2	Fu, J. *et al.*	Yes	Male
Child 3	Pt14_R	2.3	Fu, J. *et al.*	Yes	Male
Child 4	Pt21_R	2.6	Fu, J. *et al.*	Yes	Female
Child 5	Pt19_R	3.3	Fu, J. *et al.*	Yes	Male
Child 6	Pt17_R	5.4	Fu, J. *et al.*	Yes	Male
Child 7	Pt25_R	9.32	Fu, J. *et al.*	Yes	Male
Adult 1	D207	23	Meng, W. *et al.*	No	Male
Adult 2	D181	46	Meng, W. *et al.*	No	Male
Adult 3	D182	46	Meng, W. *et al.*	No	Male
Adult 4	D149	55	Meng, W. *et al.*	No	Male
Adult 5	D168	56	Meng, W. *et al.*	No	Female
Adult 6	D145	58	Meng, W. *et al.*	No	Male

Supplemental Table 1. Subject names, ages, original sources and immunosuppression status.

Name	Unique sequence count	Copy number count	Sample count	Clone count	Tissues
Child A	7,884	71,979	2	190	Lung
Child B	21,872	140,129	4	7,082	LLN, Lung
Child C	18,818	71,703	4	9,005	LLN, Lung
Child D	16,249	87,155	4	7,831	LLN, Lung
Child E	54,614	361,730	9	28,541	LLN, Lung
Child F	32,441	213,222	4	12,008	LLN, Lung
Child G	61,515	409,519	12	22,711	LLN, Lung
Child H	10,704	60,502	4	5,160	LLN, Lung
Adult A	35,999	296,105	4	11,259	LLN, Lung
Adult B	25,046	183,412	4	8,109	LLN, Lung
Adult C	29,113	242,996	4	6,639	LLN, Lung
Child 1	355,050	1,840,149	51	108,162	Blood, Colon, MLN, Ileum, BM, Duodenum, Jejunum
Child 2	168,967	867,106	23	100,877	Blood, MLN, Ileum, BM, Duodenum, Colon
Child 3	70,574	196,253	18	55,615	Blood, MLN, Ileum
Child 4	171,795	1,015,707	35	75,824	Blood, Ileum, MLN, Duodenum, Colon
Child 5	341,868	2,479,972	48	138,315	Blood, Colon, MLN, Ileum, BM, Duodenum
Child 6	159,647	1,005,215	20	62,282	Blood, MLN, Colon, Ileum
Child 7	9,432	42,078	2	3,885	Blood, Ileum
Adult 1	1,790,626	18,683,363	214	523,709	Blood, BM, SPL, Ileum, MLN, Colon, Jejunum, Lung
Adult 2	699,617	6,754,284	94	203,011	Blood, MLN, Colon, Ileum, SPL, BM, Jejunum, Lung
Adult 3	84,868	817,965	39	20,405	Blood, BM, Colon, Ileum, Jejunum, Lung, MLN, SPL
Adult 4	95,775	1,663,130	42	13,930	Blood, BM, Colon, Ileum, Jejunum, Lung, MLN, SPL
Adult 5	69,202	783,942	38	16,603	Blood, Ileum, Jejunum, Colon, SPL, BM, Lung, MLN
Adult 6	168,159	1,685,670	42	45,681	Blood, BM, Colon, Ileum, Jejunum, Lung, MLN, SPL

Supplemental Table 2. Subject sequencing and tissue data. BM, bone marrow; MLN, mesenteric lymph node; LLN, lung lymph node; SPL, spleen. In the original datasets, blood is called PBMC or PBL.

Figure	Panel	Clone type	Datapoints omitted
4	A, B	Trunk/Mutated	**LLN** (Child B, Child C); **Lung** (Child A, Child B)
5	A	Pre-trunk	**LLN** (Child B, Child C, Child H); **Lung** (Child A, Child B, Child D, Adult A, Adult B)
5	A	Trunk	**Blood** (Child 3); **LLN** (Child B, Child C, Child D); **Lung** (Child A, Child B)
5	B	Unmutated	**Blood** (Child 3, Adult 3, Adult 5, Adult 6); **LLN** (Child B, Child C, Child D, Child H, Adult A, Adult B, Adult C); **Lung** (Child D, Adult A, Adult 4, Adult 6, Adult B, Adult C); **Ileum** (Adult 4, Adult 5)
5	B	Pre-trunk	**Blood** (Child 3, Child 6, Adult 3, Adult 4, Adult 6); **LLN** (Child B, Child C, Child D, Child E, Child F, Child G, Child H, Adult A, Adult B, Adult C); **Lung** (Child A, Child B, Child C, Child D, Child E, Child F, Child H, Adult A, Adult B, Adult C)
5	B	Trunk	**Blood** (Child 3); **LLN** (Child B, Child C, Child D, Child F, Child G, Child H); **Lung** (Child A, Child B, Child C, Child D); **Ileum** (Child 3)
5	C	Pre-trunk	**LLN** (Child B, Child C, Child H); **Lung** (Child A, Child B, Child D, Adult A, Adult B)
5	C	Trunk	**Blood** (Child 3); **LLN** (Child B, Child C, Child D); **Lung** (Child A, Child B)

Supplemental Table 3. Datapoints omitted from figures due to having fewer than 10 clones. **Figure 5B** has an additional layer of filtering due to filtering clonal lineages for having at least five populated internal nodes, which is a strict filter (necessary to prevent results from being skewed by simple lineages) and results in many datapoints being omitted due to having fewer than 10 clones. LLN - lung lymph node.
